# Conserved phosphorylation events in ESRRB integrate diverse signaling pathways to govern the fate of embryonic stem cells

**DOI:** 10.3389/fcell.2026.1919736

**Published:** 2026-10-12

**Authors:** Haojie Duan, Qian Wang, Yuping Suo, Jin-An Zhou, Xiaojuan Liu, Yin Fang

**Affiliations:** 1 Departments of Laboratory Medicine, Obstetrics & Gynecology and Pediatrics, Cancer Center, Key Laboratory of Birth Defects and Related Diseases of Women and Children, Ministry of Education, Development and Related Diseases of Women and Children Key Laboratory of Sichuan Province, West China Second University Hospital, Sichuan University, Chengdu, Sichuan, China; 2 Shanxi Provincial People’s Hospital, Taiyuan, Shanxi, China; 3 Department of Blood Transfusion, Tongji Hospital, Tongji Medical College, Huazhong University of Science and Technology, Wuhan, China

**Keywords:** differentiation, ESRRB, GSK3β, protein degradation, P-TEFb, stem cell

## Abstract

Embryonic stem cells are self-renewing, pluripotent cells, normally maintained by several pluripotency transcription factors. To respond to diverse differentiation and stress signaling pathways, the undifferentiated cell state must be destabilized before embryonic stem cells can properly interpret external signals to decide the cell fate. It remains incompletely understood how diverse signals promote the exit of the self-renewal state. Here, we show that both acute DNA damage and diverse differentiation signaling pathways converge at the proteasomal destruction of Estrogen-related receptor β (ESRRB), a key pluripotency transcription factor. Environmental agents such as UV light and genotoxic chemicals activate P-TEFb protein kinase to directly phosphorylate ESRRB to promote its proteasomal degradation. On the other hand, diverse differentiation signaling pathways also promote ESRRB degradation. Interestingly, the responsible kinase is GSK3β, instead of P-TEFb, in these biological contexts. Substituting five Ser residues in ESRRB with Ala confers resistance to GSK3β-dependent ESRRB degradation and compromises cell differentiation. These results demonstrate that conserved post-translational modifications in ESRRB integrate diverse signaling pathways to govern the fate of embryonic stem cells.

## Introduction

Pluripotent stem cells, such as embryonic stem cells (ESCs) and induced pluripotent stem cells (iPSCs), have several distinct properties rendering them attractive in basic research and medicine (Yamanaka, 2026). They are characterized by an unlimited self-renewal potential, that is to proliferate extensively in an undifferentiated cell state (Evans, 2011). Another defining feature is that they are pluripotent, that is, they are responsive to a variety of inductive signaling pathways to differentiate and generate most cell types (Nishikawa et al., 2008). In addition, pluripotent stem cells are also highly sensitive to genotoxic chemicals, presumably as a cellular mechanism to avoid passing damaged genomes on to differentiated cells. This exquisite sensitivity is thought to account for the unusual success of platinum-based drugs in treating germ cell tumors, the malignant counterparts of pluripotent stem cells (Oosterhuis and Looijenga, 2019).

Transcription factors are central regulators of these features in pluripotent stem cells. POU5F1 (POU class 5 homeobox 1, commonly known as OCT4) and SOX2 (SRY-box transcription factor 2) are essential transcription factors to keep ESCs in an undifferentiated state, and required to reprogram differentiated cells to iPSCs (Takahashi and Yamanaka, 2006; Takahashi et al., 2007). These master pluripotency transcription factors maintain the identity of pluripotent stem cells by simultaneously activating downstream factors required for self-renewal and repressing the transcriptional programs required for differentiation (Young, 2011). A second class of transcription factors, such as NANOG (Nanog homeobox) and ESRRB (Estrogen-related receptor β), seem to reinforce the identity of pluripotent stem cells. Under optimal culture conditions in favor of self-renewal, their functions are dispensable (Chambers et al., 2007). Mouse ESCs can proliferate normally without any signs of differentiation when *Esrrb* is genetically ablated. However, *Esrrb*-null mouse ESCs are prone to differentiation under suboptimal culture conditions. Conversely, overexpression of *Esrrb* in mouse ESCs can partly block differentiation (Festuccia et al., 2018). In the context of genotoxic insults, we recently demonstrated that ESRRB protein is rapidly degraded by the proteosome. Various genotoxic stressors, such as ultraviolet irradiation, cisplatin and doxorubicin, converge at the activation of the P-TEFb protein kinase to directly phosphorylate five serine residues in ESRRB protein (S27, S39, S63, S199 and S207). Treating mouse ESCs with small-molecular inhibitors of P-TEFb can fully block ESRRB protein degradation. Consistently, mutating all five serine residues to alanine (5A mutant) also prevents ESRRB protein degradation triggered by genotoxic stressors (Fang et al., 2022).

However, in the context of ESC differentiation, it remains poorly understood how ESRRB protein is regulated and how its dysregulation affects cell differentiation. In the present study, we show that, in a variety of mouse ESC differentiation models, ESRRB protein is rapidly degraded. Interestingly, ESRRB 5A mutant can resist degradation, resulting in compromised differentiation. The responsible kinase is GSK3β instead of P-TEFb. Thus, conserved post-translational modifications in ESRRB protein integrate diverse signaling pathways to govern the fate of embryonic stem cells.

## Results

### Genotoxic insults trigger ESRRB protein degradation

Previously, we have shown that several genotoxic insults can trigger ESRRB protein degradation, when mouse embryonic stem cells (mESCs) were cultured using serum plus leukemia inhibitory factor (LIF) condition (Fang et al., 2022). In the present study, we first investigated whether this phenomenon still holds when mESCs were grown in the 2i condition, that is, when MEK and GSK3β kinase activities are inhibited by chemical inhibitors. mESCs cultured in the 2i condition are termed in a naïve or ground state, as they are developmentally more primitive than those cultured in traditional serum plus LIF condition (Marks et al., 2012). For the simplicity, we will refer the serum plus LIF condition as “-2i” condition.

mESCs cultured in the 2i condition were treated with several genotoxic stressors, including ultraviolet irradiation (Figure 1A), doxorubicin (Figure 1B), cisplatin (Figure 1C). These treatments induced different types of acute DNA damage, but they all reduced the protein expression of ESRRB. Co-treatment with the proteasomal inhibitor MG132 efficiently recovered ESRRB expression, indicating that genotoxic stressors induce the proteasomal degradation of ESRRB. Most cellular P-TEFb (CDK9/cyclin T) is sequestered and inactivated by 7SK snRNP, composed of 7SK snRNA, LARP7 and HEXIM1/2 proteins (Li et al., 2005). All of these genotoxic stressors can activate P-TEFb kinase activity, by releasing P-TEFb from the 7SK snRNP (Fang et al., 2022). Co-treatment with flavopiridol, a chemical inhibitor of P-TEFb (Chao and Price, 2001), also prevented the downregulation of ESRRB by genotoxic stressors (Figures 1D–F). Of note, treatment with flavopiridol alone can also release P-TEFb from the 7SK snRNP, but simultaneously inhibits P-TEFb kinase activity. Consistently, treatment with flavopiridol alone did not alter ESRRB expression. Lastly, we examined FLAG-tagged wild-type and 5A mutant ESRRB reconstituted in *Esrrb*-null mESCs. 5A mutant contains alanine substitution of all five serine residues (S27, S39, S63, S199 and S207) in ESRRB protein (NP_001152972), and cannot be phosphorylated by P-TEFb (Fang et al., 2022). FLAG-tagged wild-type ESRRB protein, like its endogenous counterpart, was efficiently degraded by genotoxic stressors. In contrast, FLAG-tagged 5A mutant ESRRB was minimally affected (Figures 1G–I). Thus, in both -2i and 2i self-renewal conditions, genotoxic stressors activate P-TEFb protein kinase to phosphorylate ESRRB protein, triggering its proteasomal degradation. These observations prompted us to investigate whether other biological contexts would modulate ESRRB expression. And if so, which signaling is involved, and how it would affect ESC differentiation.

**FIGURE 1:**
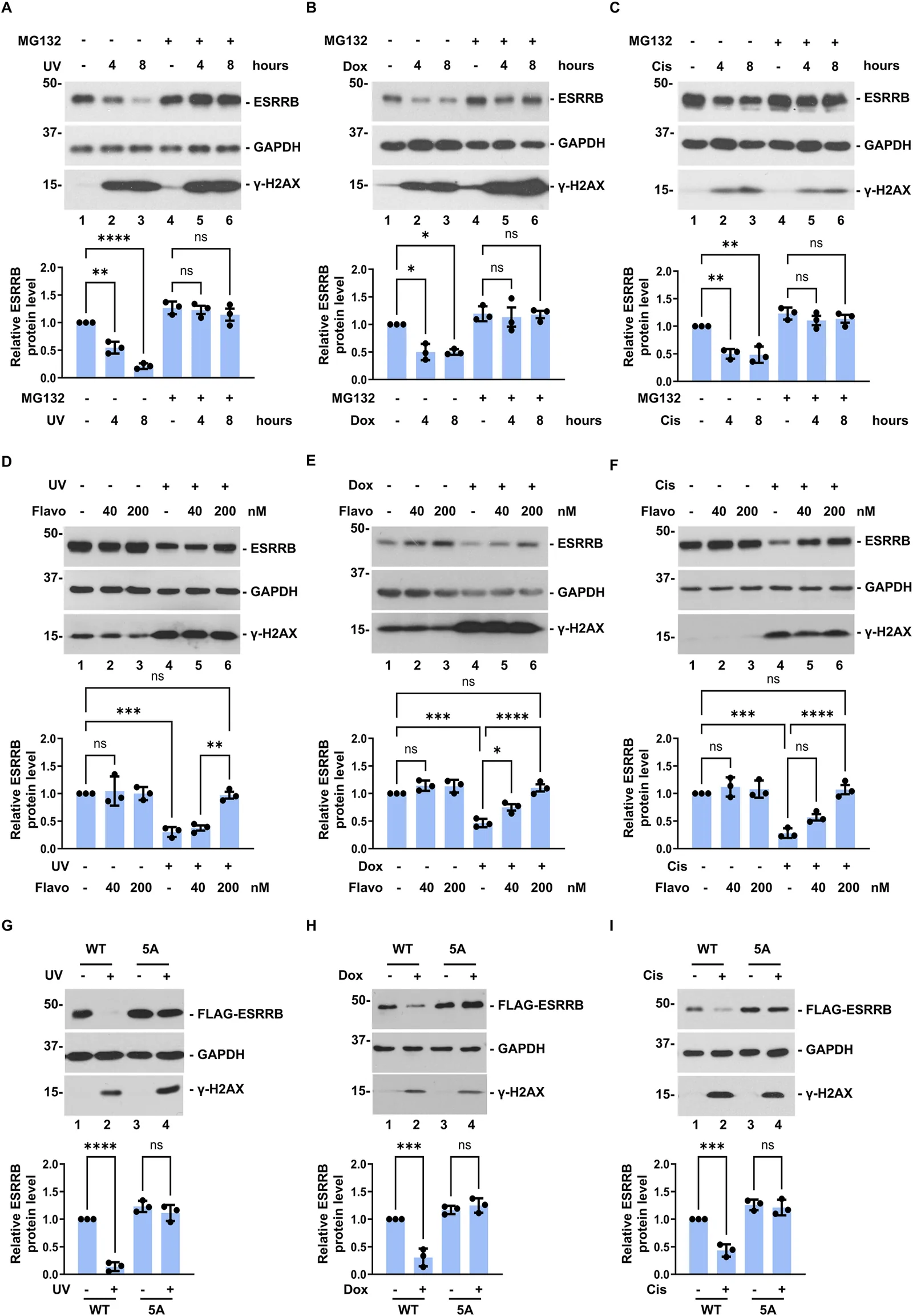
Genotoxic insults trigger ESRRB protein degradation. **(A–C)** DNA damage induces protein degradation of ESRRB in R1/E mESC cells. Western blot analyses of indicated proteins from total cell lysates derived from R1/E mESC cells, treated with three different types of DNA damage, including ultraviolet radiation (UV,10 J/m^2^) **(A)**, doxorubicin (Dox, 1 μM) **(B)**, or cisplatin (Cis, 5 μM) **(C)**, in the absence or presence of the proteasomal inhibitor, MG132 (1 μM). Quantification of ESRRB protein levels is shown below the corresponding Western blots. ESRRB levels were normalized to GAPDH, with the untreated control set to 1. **(D–F)** P-TEFb inhibitor, flavopiridol (Flavo) blocks DNA damage-induced ESRRB protein degradation. Western blot analyses of indicated proteins from total cell lysates derived from R1/E mESC cells, treated with UV **(D)**, doxorubicin (Dox) **(E)**, or cisplatin (Cis) **(F)** in the absence or presence of flavopiridol (Flavo, 40 or 200 nM). Quantification of ESRRB protein levels is shown below the corresponding Western blots. ESRRB levels were normalized to GAPDH, with the untreated control set to 1. **(G–I)** 5A mutant ESRRB (S27, S39, S63, S199 and S207 mutated to alanine) resists DNA damage-induced protein degradation. Western blot analysis of indicated proteins from total cell lysates of Esrrb-null R1/E mESC cells reconstituted with FLAG-tagged wild-type (WT) or 5A mutant ESRRB (5A), treated with UV **(G)**, doxorubicin (Dox) **(H)**, or cisplatin (Cis) **(I)**. Quantification of ESRRB protein levels is shown below the corresponding Western blots. ESRRB levels were normalized to GAPDH, with the untreated control set to 1.

### Differentiation of mESC to epiblast stem cells (EpiSCs) triggers ESRRB protein degradation

Mouse embryonic stem cells (mESCs) cultured in the presence of 2i and LIF represent a naive pluripotency state. Withdrawing these factors triggers differentiation toward epiblast stem cells (EpiSCs) (Buecker et al., 2014). ESRRB expression was significantly downregulated in both wild-type (WT) mESCs. FLAG-tagged wild-type ESRRB (EKO-WT) reconstituted in *Esrrb*-null mESCs (EKO) was also decreased to a similar degree as its endogenous counterpart. In contrast, the expression of FLAG-tagged 5A ESRRB reconstituted in *Esrrb*-null mESCs (EKO-5A) was minimally affected by 2i and LIF removal (Figure 2A). We assessed the effect of ESRRB degradation in the course of mESC differentiation. Pecam1 is highly expressed on the cell surface of mESCs, and widely used as an undifferentiated marker (Robson et al., 2001). To track differentiation kinetics, we performed flow cytometry to analyze Pecam1 expression 3 days post-2i and LIF withdrawal. As expected, nearly 100% of cells across all groups, including wild-type (WT), Esrrb knockout (EKO), EKO-WT, and EKO-5A, were Pecam1 positive (Pecam1^+^) under the 2i/LIF condition. Three days after 2i and LIF removal, the percentage of Pecam1^+^ cells decreased to 54.9% in WT cells and similarly 53.5% in EKO-WT cells. Less Pecam1^+^ cells (48.2%) were left in EKO cells, consistent with a role of ESRRB in maintaining the undifferentiated states. Notably, 68.7% of EKO-5A cells remained Pecam1^+^ (Figure 2B), suggesting that ESRRB-5A mutant inhibits the differentiation of mESCs into EpiSCs.

**FIGURE 2:**
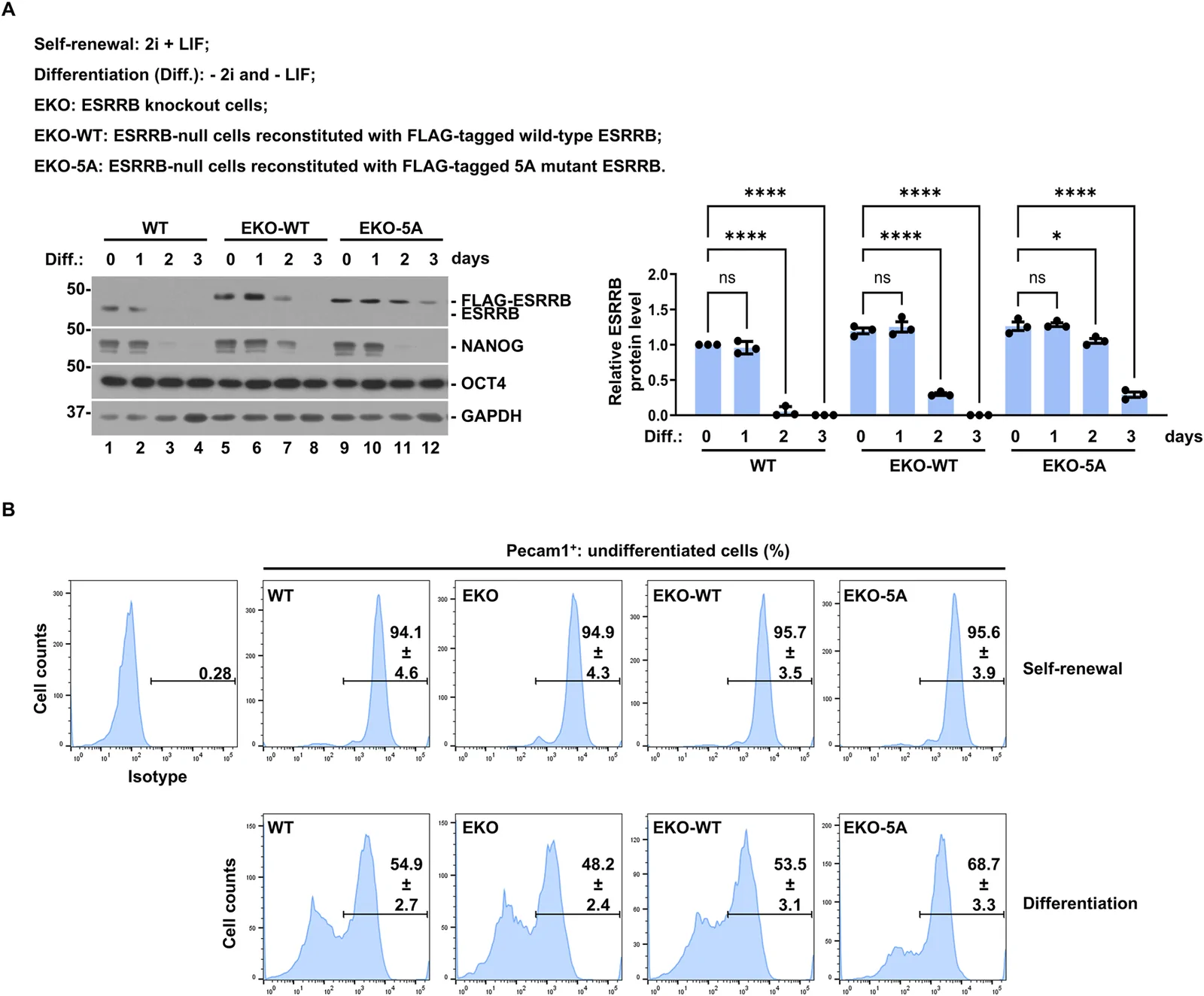
Differentiation of mESCs to epiblast stem cells (EpiSCs) triggers ESRRB protein degradation. **(A)** Western blot analysis of indicated proteins in wild type R1/E mESCs (WT), Esrrb-null cells reconstituted with FLAG-tagged wild-type (EKO-WT), or Esrrb-null cells reconstituted with 5A mutant ESRRB (EKO-5A) during epiblast stem cell (EpiSC) differentiation. Removal of 2i and LIF triggers EpiSC differentiation from mESC. Total cell lysates were generated at day 0 (self-renewal), and 1, 2 or 3 days after differentiation. Quantification of ESRRB protein levels is shown beside the corresponding Western blots. ESRRB levels were normalized to GAPDH, with the untreated control set to 1. **(B)** Analysis of Pecam1-positive cells by flow cytometry in WT, EKO, EKO-WT, or EKO-5A mESCs 3 days after differentiation. Pecam1-positive cells denote undifferentiated cells. Data are shown as mean ± SD (n = 3)

### Spontaneous differentiation of mESCs triggers ESRRB protein degradation

mESCs cultured in the -2i condition are considered in a primed state, and removing LIF initiates spontaneous mESC differentiation. FLAG-tagged wild-type ESRRB reconstituted in *Esrrb*-null mESCs was also downregulated by LIF removal. In contrast, the expression of FLAG-tagged 5A ESRRB reconstituted in *Esrrb*-null mESCs was minimally affected by LIF removal (Figure 3A).

**FIGURE 3:**
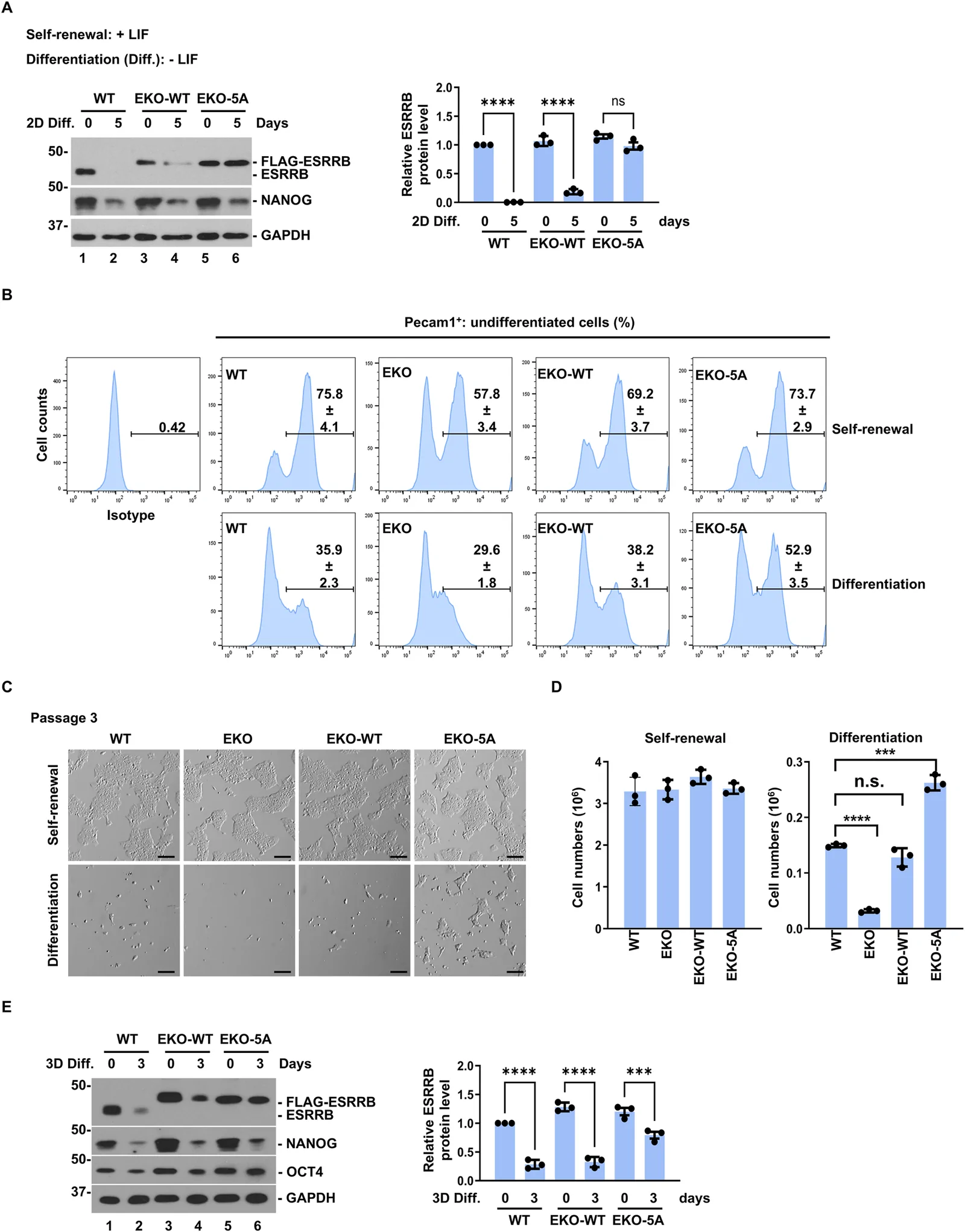
Spontaneous differentiation of mESCs triggers ESRRB protein degradation. **(A)** Western blot analysis of indicated proteins in wild type R1/E mESCs (WT), Esrrb-null cells reconstituted with FLAG-tagged wild-type (EKO-WT), or Esrrb-null cells reconstituted with 5A mutant ESRRB (EKO-5A) during the course of mESC differentiation. Total cell lysates were generated at day 0 (self-renewal) and day 5 after differentiation (LIF removed). Quantification of ESRRB protein levels is shown beside the corresponding Western blots. ESRRB levels were normalized to GAPDH, with the untreated control set to 1. **(B)** Analysis of Pecam1-positive cells by flow cytometry in WT, EKO, EKO-WT, or EKO-5A mESCs 3 days after differentiation. Pecam1-positive cells denote undifferentiated cells. **(C)** Clonal survival assay indicative of undifferentiated cells during differentiation. Cells under self-renewal or differentiation condition were passaged three times (Passage 3), and representative phase-contrast images of surviving mESCs from three independent experiments were shown. **(D)** Quantification of surviving clones in **(C)**. Data are presented as mean ± SD from three independent experiments. ***, p < 0.001; n. s not significant; (one-way ANOVA). **(E)** Western blot analysis of indicated proteins in wild-type R1/E mESCs (WT) and Esrrb-null cells reconstituted with either FLAG-tagged wild-type ESRRB (EKO-WT) or the 5A mutant (EKO-5A) after 3 days of embryoid body (EB) differentiation. Quantification of ESRRB protein levels is shown beside the corresponding Western blots. ESRRB levels were normalized to GAPDH, with the untreated control set to 1.

We assessed the effect of ESRRB degradation in the course of mESC differentiation. We used flow cytometry to analyze Pecam1 expression 3 days after LIF removal. As expected, nearly 70% of wild-type cells were positive for Pecam1 expression (Pecam1^+^) in the -2i condition. Similar situation was also observed in *Esrrb*-null cells reconstituted with FLAG-tagged wild-type ESRRB (EKO-WT), and *Esrrb*-null cells reconstituted with FLAG-tagged 5A ESRRB (EKO-5A), whereas the portion was lower in *Esrrb*-null cells (EKO). After LIF removal for 3 days, Pecam1^+^ cells decreased to 35.9% in wild-type cells, and similarly 38.2% in *Esrrb*-null cells reconstituted with FLAG-tagged wild-type ESRRB. In contrast, only 29.6% of *Esrrb*-null cells were Pecam1^+^, suggesting that losing Esrrb expression accelerates ESC differentiation. Consistently, in *Esrrb*-null cells reconstituted with FLAG-tagged 5A mutant ESRRB, a higher portion of Pecam1^+^ cells was observed (Figure 3B). These results suggest that a timely degradation of ESRRB protein facilitates ESC differentiation.

In addition, we used orthogonal methods to determine the degree of differentiation. Firstly, we measured surviving mESCs after serial passages in the differentiation condition. Cells were dissociated by trypsin after 2 days of LIF removal, and replated into fresh medium without LIF (passage 1). After 2 days, the same procedure was repeated twice (passage 2 and passage 3). Importantly, a significantly more clones could be detected in *Esrrb*-null cells reconstituted with 5A mutant ESRRB than in wild-type cells or in *Esrrb*-null cells reconstituted with wild-type ESRRB (Figures 3C,D). Second, Embryoid bodies are three-dimensional aggregates of multiple cell types, formed by culturing mESCs in suspension. The assembly of embryoid bodies *in vitro* recapitulates many aspects of cell differentiation during early embryogenesis *in vivo*, and is frequently used as an initial step to generate more differentiated cell types (Daley, 2003). After 3 days, embryoid bodies were harvested for protein blot analysis. The expression levels of ESRRB in wild-type and FLAG-tagged wild-type ESRRB in *Esrrb*-null cells were reduced. In contrast, FLAG-tagged 5A mutant ESRRB expression was only moderately decreased (Figure 3E). Taken together, these results suggest that differentiation signaling promotes ESRRB degradation to facilitate the exit from ESC self-renewal. Conversely, a delay in the degradation of 5A mutant ESRRB compromises cell differentiation.

### Differentiation of mESCs to neural progenitor cells (NPCs) triggers ESRRB protein degradation

Retinoic acid is a signaling molecule for cell differentiation *in vivo*, and frequently used to differentiate mESCs *in vitro* (Brown, 2023). To initiate differentiation, the -2i medium was replaced with fresh medium supplemented with 50 nM retinoic acid without LIF. After 3 days, cells were harvested for protein blot analysis. The expression levels of endogenous ESRRB in wild-type and FLAG-tagged wild-type ESRRB in *Esrrb*-null cells was reduced. In contrast, FLAG-tagged 5A mutant ESRRB expression were only moderately decreased (Figure 4A). To assess the effect of ESRRB-5A mutant on preventing differentiation, mESCs were dissociated into single cells and seeded at a density of 600 cells per well. Cells were then cultured in LIF-deficient medium supplemented with retinoic acid (-LIF + RA) to induce differentiation. Following 6 days of culture, alkaline phosphatase (AP) staining was performed. Notably, EKO-5A cells yielded significantly more AP-positive colonies compared to the WT or EKO-WT cells (Figures 4B,C), indicating ESRRB-5A mutant conferred resistance to RA-induced differentiation. Secondly, we used a more defined condition to induce neural progenitor differentiation. mESCs were cultured in naïve medium (N2B27 with 2i and LIF) (Ying et al., 2003). To initiate NPC differentiation, 2i and LIF were removed. After 3 or 5-day differentiation, the expression levels of ESRRB in wild-type and FLAG-tagged wild-type ESRRB were reduced. In contrast, FLAG-tagged 5A mutant ESRRB expression was only minimally decreased (Figure 4D). Thus, mESCs differentiation towards neural progenitor cells (NPCs) triggers ESRRB protein degradation.

**FIGURE 4:**
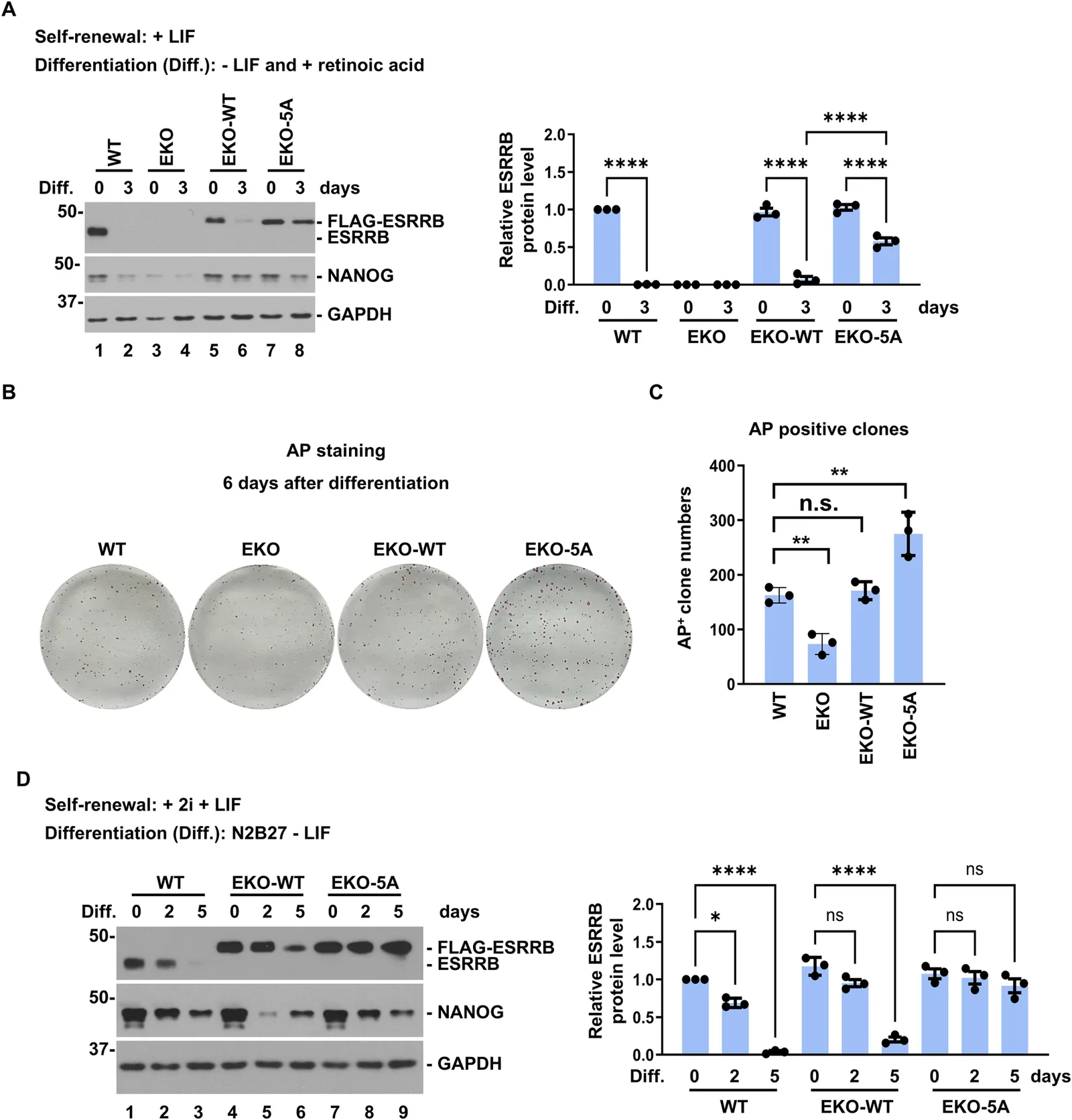
Differentiation of mESCs to neural progenitor cells (NPCs) triggers ESRRB protein degradation. **(A)** Western blot analysis of indicated proteins in wild type R1/E mESCs (WT), Esrrb-null cells reconstituted with FLAG-tagged wild-type (EKO-WT), or Esrrb-null cells reconstituted with 5A mutant ESRRB (EKO-5A) during the course of mESC differentiation. Total cell lysates were generated at day 0 (self-renewal), and 3 days after differentiation (-LIF/+RA). Quantification of ESRRB protein levels is shown beside the corresponding Western blots. ESRRB levels were normalized to GAPDH, with the untreated control set to 1. **(B)** Alkaline phosphatase (AP) staining of surviving undifferentiated R1/E mESCs after 6 days of differentiation. Representative images of three independent experiments were shown. **(C)** Quantification of AP positive clones in **(B)** The bar plot represents mean ± SD (n = 3). **, p < 0.01; n. s not significant; (one-way ANOVA). **(D)** Western blot analysis of indicated proteins in wild type R1/E mESCs (WT), Esrrb-null cells reconstituted with FLAG-tagged wild-type (EKO-WT), or Esrrb-null cells reconstituted with 5A mutant ESRRB (EKO-5A) under defined neural progenitor differentiation condition. Quantification of ESRRB protein levels is shown beside the corresponding Western blots. ESRRB levels were normalized to GAPDH, with the untreated control set to 1.

### Teratoma formation from mESCs growing in the +2i condition

Teratoma formation is an *in vivo* assay to monitor the pluripotency of ESCs (Zhang et al., 2008a). In the +2i condition *in vitro*, that is, the optimal culturing condition in favor of self-renewal, the rate of cell proliferation was comparable in wild-type mESCs, *Esrrb*-null mESCs, and *Esrrb*-null mESCs reconstituted with wild-type or 5A mutant ESRRB (Figure 5A). When inoculated into immunocompromised NSG mice, mESCs can form teratomas, comprised of both undifferentiated cells and differentiated cells. Teratomas formed by *Esrrb*-null mESCs were the smallest in size. The sizes of teratomas formed by wild-type mESCs and *Esrrb*-null mESCs reconstituted with wild-type ESRRB were comparable, and greater than those formed by *Esrrb*-null mESCs. Teratomas formed by *Esrrb*-null mESCs reconstituted with mutant 5A ESRRB were the largest (Figures 5B,C). Histological analysis by H&E staining further revealed teratomas containing differentiated tissues from three germ layers, in teratomas from each group, with no obvious undifferentiated regions (Figure 5D). Because the size of the teratoma is proportional to the proliferative potential of mESCs, EKO-5A cells consistently generated significantly more surviving colonies than WT or EKO-WT cells (Figures 3C,D) under continuous differentiation conditions following LIF withdrawal, cells were serially passaged for three passages. EKO-5A cells produced significantly more AP-positive colonies than WT or EKO-WT cells (Figures 4B,C). These findings further support a role for ESRRB-5A in promoting mESC maintenance while preserving pluripotent differentiation potential. Taken together, these *in vitro* and *in vivo* findings support a role for ESRRB-5A in promoting the maintenance of the self-renewing state of mESCs.

**FIGURE 5:**
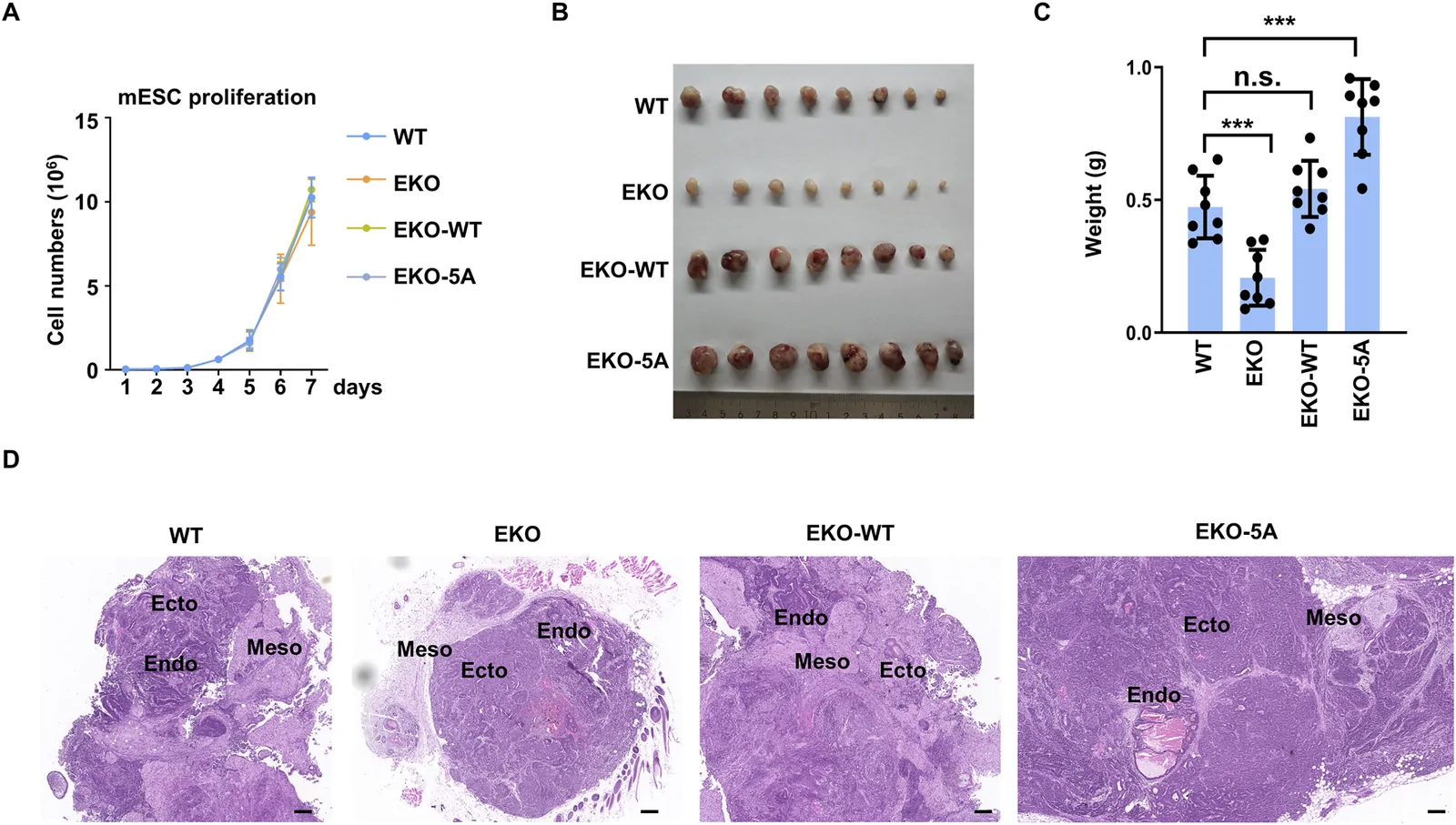
Teratoma formation assay *in vivo*. **(A)** Comparison of proliferation rates among wild-type (WT), Esrrb-null (EKO), and Esrrb-null mESCs reconstituted with either FLAG-tagged wild-type ESRRB (EKO-WT) or the 5A mutant (EKO-5A). **(B)** Representative images of teratomas derived from wild-type (WT), Esrrb-null, Esrrb-null mESCs reconstituted with FLAG-tagged wild-type (EKO-WT) or 5A mutant ESRRB (EKO-5A) mESCs subcutaneously injected into immunocompromised NSG mice. **(C)** Quantification of teratoma weight in **(B)**. Data are presented as mean ± SD from n = 8 mice per group. ***, p < 0.001; n. s not significant; (one-way ANOVA). **(D)** Representative of H&E staining of teratoma sections derived from wild-type (WT), Esrrb-null, Esrrb-null mESCs reconstituted with FLAG-tagged wild-type (EKO-WT) or 5A mutant ESRRB (EKO-5A) mESCs. Teratomas containing differentiated tissues from three germ layers. Scale bars, 50 μm. Ecto, ectoderm, Meso, mesoderm, Endo, endoderm.

### Differentiation-triggered ESRRB degradation is dependent on GSK3β kinase activity

We have shown that ESRRB protein degradation induced by genotoxic stressors is dependent on P-TEFb kinase activity (Figure 1). However, co-treatment with P-TEFb chemical inhibitor flavopiridol did not block differentiation-induced ESRRB protein degradation (Figure 6A). P-TEFb is comprised of CDK9 catalytic subunit and cyclin T regulatory subunit. CDK family members can phosphorylate their substrates at a consensus motif, that is threonine or serine followed by a proline residue (S/TP site). Given cell proliferation rate may be coupled with cell differentiation, we examined whether CDK1, CDK2, CDK4 and CDK6, key regulators of cell cycle progression, might be involved. However, co-treatment with chemical inhibitors of CDK1/2 (RO3306) (Figure 6B), or CDK4/6 (Palbociclib) (Figure 6C) had no effect on differentiation-induced ESRRB protein degradation. MEK and GSK3β kinase signaling pathways are known to promote mESC differentiation. We found that co-treatment with a MEK inhibitor (MEKi, PD0325901) did not prevent ESRRB protein degradation (Figure 6D). In contrast, co-treatment with a GSK3β inhibitor (GSK3βi, CHIR99021) largely blocked ESRRB protein degradation (Figure 6E). To investigate whether the five serine residues of ESRRB conform to the GSK3β recognition consensus, we performed a detailed sequence alignment of the regions flanking these residues. This analysis revealed that Ser63 is followed by a serine residue at the +4 position (Ser63-X-X-X-Ser67), which perfectly matches the canonical GSK3β priming motif S/TXXXS/T. To determine whether GSK3β directly phosphorylates ESRRB, we performed *in vitro* kinase assays using bacterially expressed and purified His-tagged wild-type or 5A mutant ESRRB proteins as the substrate and recombinant GST-tagged GSK3β as the kinase. Wild-type ESRRB was readily phosphorylated by GSK3β, whereas the 5A mutant was not (Figure 6F). To investigate whether GSK3β promotes ESRRB ubiquitination, we analyzed the ubiquitination status of wild-type and 5A mutant ESRRB upon differentiation. EKO-WT, EKO-5A, and EKO cells transfected with HA-ubiquitin were cultured in 2i/LIF and then induced to differentiate by LIF/PD0325901 withdrawal for 3 days, with or without CHIR99021. CHIR99021 withdrawal led to a modest increase in ubiquitination of wild-type ESRRB, whereas the 5A mutant did not undergo ubiquitination under all conditions (Figure 6G). Collectively, these data indicate that GSK3β-dependent phosphorylation of ESRRB promotes its ubiquitination during mESC differentiation. To evaluate whether GSK3β inhibition affects differentiation through ESRRB stabilization, we compared the differentiation behavior of WT, EKO, EKO-WT, and EKO-5A cells upon LIF/PD0325901 withdrawal in the presence of CHIR99021. Analysis of pecam1 by flow cytometry showed that CHIR99021 delayed differentiation, albeit to variable degrees in WT, EKO, EKO-WT and EKO-5A, consistent with MEK inhibition being the predominant differentiation trigger. Notably, EKO-5A cells displayed comparable differentiation to EKO-WT cells in the presence of CHIR99021 (Figure 6H), whereas they showed reduced differentiation relative to EKO-WT cells without CHIR99021 (Figure 2B). Additionally, CHIR99021 treatment partially attenuated differentiation of EKO-5A cells (Figures 2B, 6H; 68.7 %± 3.3% vs. 72.3% ± 3.5%). Collectively, these data indicate that CHIR99021 primarily acts by preventing GSK3β-dependent ESRRB degradation, thereby maintaining the undifferentiated state. Consistently, a greater number of alkaline phosphatase-positive clones were detected by GSK3βi treatment *versus* vehicle treatment, indicating that GSK3β kinase activity is required for mESC differentiation. These observations suggest that GSK3β facilitates mESC differentiation, at least partly, by promoting ESRRB protein degradation.

**FIGURE 6:**
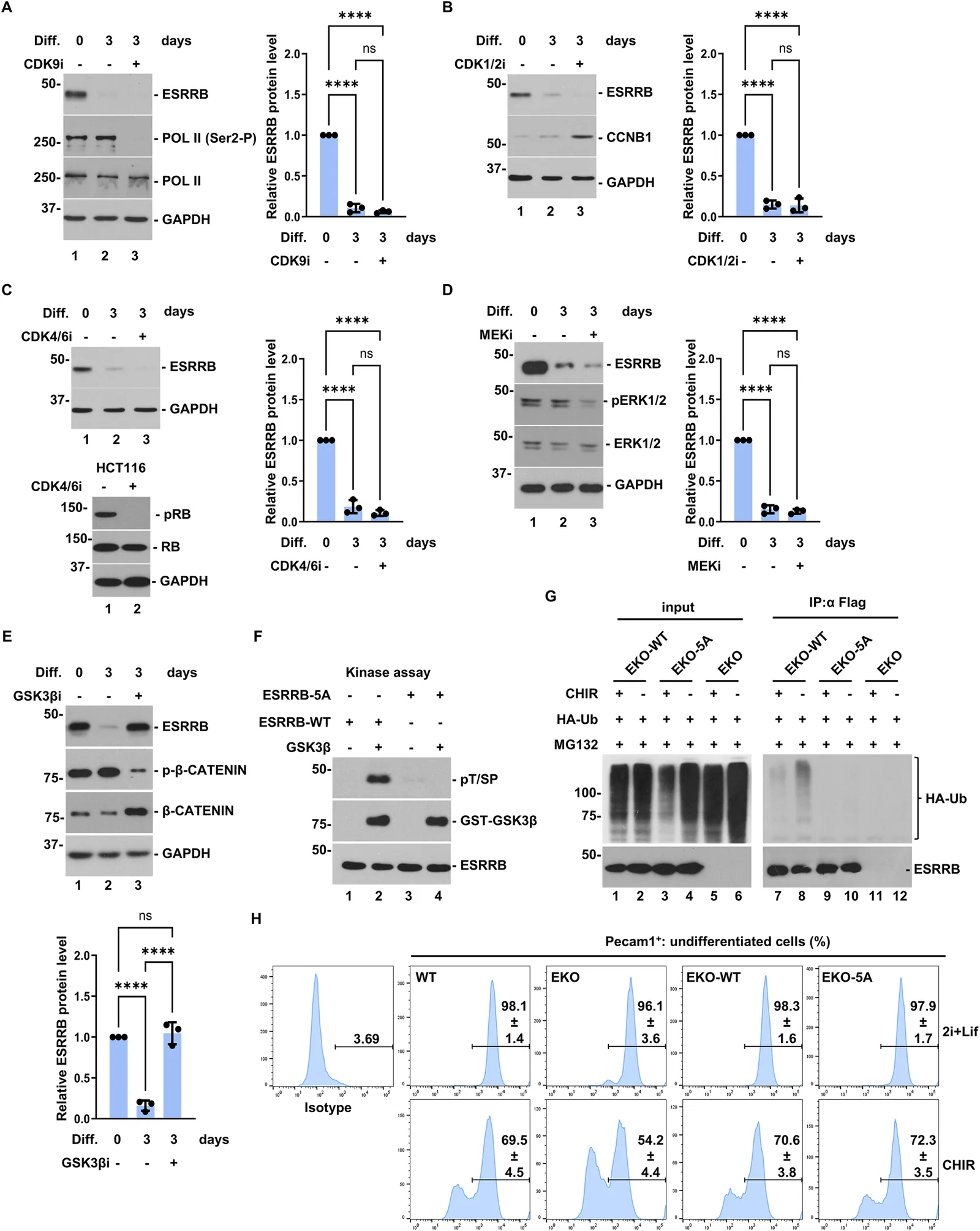
Differentiation-triggered ESRRB degradation is dependent on GSK3β kinase activity. **(A–E)** Western blot analyses of indicated proteins from total cell lysate of R1/E ES cells, treated with inhibitor of P-TEFb (P-TEFbi, Flavo, 200 nM) **(A)**, CDK1/2 (CDK1/2i, RO3306, 3 μM) **(B)** CDK4/6 (CDK4/6i, Palbociclib, 1 μM) **(C)**, MEK (MEKi, PD0325901, 1 μM) **(D)** or GSK3β (GSK3βi, CHIR99021, 3 μM) **(E)** cultured in the absence or presence of differentiation medium (-LIF + RA) for 3 days. In **(C)**, Western blot analysis of total RB and phosphorylated RB (p-RB) in HCT116 cells treated with palbociclib is additionally shown as a positive control to validate CDK4/6 inhibitor activity. Quantification of ESRRB protein levels is shown beside or below the corresponding Western blots. ESRRB levels were normalized to GAPDH, with the untreated control set to 1. **(F)** GSK3β kinase assay using recombinant wild-type (WT) or 5A ESRRB proteins. **(G)** Analysis of ubiquitination status of wild-type and 5A mutant ESRRB proteins upon differentiation, initiated by LIF and PD0325901 withdrawal, in the presence or absence of the GSK3β inhibitor CHIR99021. **(H)** Analysis of Pecam1-positive cells by flow cytometry in WT, EKO, EKO-WT, or EKO-5A mESCs 3 days after differentiation by withdrawal of LIF and PD0325901. Pecam1-positive cells denote undifferentiated cells. Data are shown as mean ± SD (n = 3)

## Discussion

Pluripotent stem cells, such as embryonic stem cells and induced pluripotent stem cells, serve as excellent models to investigate molecular mechanisms of stem cell self-renewal and differentiation. In addition, their abilities to differentiate into various cell types have long been viewed as potential solutions to develop cell-based therapeutics. Methodologies to culture pluripotent stem cells in a large scale are relatively well developed. In contrast, it is still a major challenge to differentiate these cells to generate homogenous, medically relevant cell types. As shown in the present study and many other previous studies, differentiation is not a synchronized process, and many pluripotent stem cells resist differentiation signals. Because the pluripotent state must first be dismantled to allow differentiation, factors enforcing self-renewal, such as Esrrb, must be removed. Early RNAi-based studies suggested that Esrrb is essential for maintaining ESC identity (Ivanova et al., 2006; Loh et al., 2006; Zhang et al., 2008a); however, subsequent genetic deletion experiments using Cre/loxP demonstrated that Esrrb is dispensable for both *in vitro* and *in vivo* ESC maintenance (Martello et al., 2012), a finding further corroborated by our own work (Fang et al., 2022 and the present study). Although Esrrb can facilitate somatic cell reprogramming to iPSCs (Adachi et al., 2018), it is not an essential reprogramming factor, as originally shown by Takahashi and Yamanaka (Takahashi and Yamanaka, 2006) and Okita et al. (Okita et al., 2007). Collectively, these observations indicate that Esrrb functions as an accessory factor that reinforces, but is not required for, the pluripotent state. Indeed, we find that Esrrb is not required under optimal self-renewal culture conditions, but its genetic deletion facilitates differentiation under various conditions. These observations suggest that removing Esrrb may facilitate a homogeneous, synchronized differentiation process. Other factors, notably Nanog, also behave similarly as Esrrb. Nevertheless, even in the absence of Esrrb or Nanog, many pluripotent stem cells are still resistant to differentiation signals, suggesting that other factors may be involved. In the present study, we also identify GSK3β as a potential kinase to promote ESRRB proteasomal degradation. It is interesting that DNA damage agents also trigger ESRRB proteasomal degradation. But in the latter context, the responsible kinase is P-TEFb (CDK9/cyclin T1). Regardless, both GSK3β and P-TEFb phosphorylate the same amino acid residues in ESRRB to trigger its proteasomal degradation. This observation indicates that diverse differentiation signals may converge at phosphorylating ESRRB to induce its degradation, thus destabilizing the self-renewal state. Importantly, this post-translational regulation is distinct from the previously described transcriptional control of Esrrb by GSK3 signaling (Martello et al., 2012). To the best of our knowledge, the convergence of DNA damage and GSK3 pathways on the same phosphorylation sites to promote ESRRB degradation is novel. Further studies are needed to identify other factors similar to ESRRB, that is, their functions are not required under optimal self-renewal culture conditions, but their absence renders pluripotent stem cells more prone to lineage-specific differentiation to generate a more homogenous cell population.

## Materials and methods

### Cell culture

R1/E mouse stem cells (mESCs) were purchased from ATCC, and verified to be *mycoplasma* free by PCR. Cells were routinely maintained as previously described (Dai et al., 2014). FLAG-tagged wild-type and 5A mutant ESRRB reconstituted in *Esrrb*-null mESCs were previously described (Fang et al., 2022). To initiate differentiation toward an epiblast stem cell (EpiSC) like state, mESCs were initially maintained in standard culture medium supplemented with 2i containing 1 μM PD0325901 (MEK inhibitor) and 3 μM CHIR99021 (GSK3β inhibitor), and leukemia inhibitory factor (LIF). Cells were then washed with phosphate buffered saline (PBS) to thoroughly remove residual factors and subsequently replaced with medium without 2i or LIF (Buecker et al., 2014). For spontaneous differentiation, mESCs were cultured in standard maintenance medium containing LIF. To initiate differentiation, the culture medium was replaced with fresh medium without LIF, and cells were allowed to differentiate for the indicated time points. For neural progenitor cell (NPC) differentiation, two protocols were used. In the first approach, mESCs maintained in standard LIF-containing medium were induced towards NPC by adding retinoic acid and withdrawing LIF. In the second approach, mESCs maintained in 2i condition were first expanded in medium consisting of a 1:1 mixture of DMEM/F12 and neurobasal medium, supplemented with 2i and LIF. Subsequently, differentiation was initiated by removing 2i and LIF (Ying et al., 2003).

### Antibodies and compounds

Following commercial antibodies were used: anti-ESRRB (R&D, PP-H6705-00,1:4,000); anti-FLAG M2 (Sigma, A8562, 1:10,000); anti-PECAM1-APC (R&D, FAB3628A, 1:50); anti-HA (Santa Cruz, sc-7392, 1:1,000); Anti-Nanog (Bethyl, A300-397A, 1:4,000); anti-Ɣ-H2AX (Abcam, ab2893, 1:1000); p-TP/SP specific antibody (Abcam, ab9344, 1:1,000); anti-Gapdh (KangChen, KC-5G5, 1:100,000); anti-RNA Pol II (Covance, 8WG16, MPY-127R, 1:2,000); RNA polymerase II Ser2-CTD (Covance, H5, MPY-129R, 1:2,000); anti-RB (Abcam, ab181616, 1:1,000); anti-p-RB (Ser807+Ser811) (Abcam, ab277774, 1:1,000); anti-β-Catenin (Santa Cruz, SC-7963, 1:200); anti- Phospho- β-Catenin (Cell Signaling Technology, 9561, 1:1000); anti-CCNB1 (Santa Cruz, SC-245, 1:200). Following commercial compounds were used: Cisplatin (MCE, HY17394, 5 μM), Doxorubicin (Sigma, D1515, 1 μM), MG132 (MCE, HY13259, 1 μM), Flavopiridol (Selleck, S1230, 200 nM), RO-3306 (Selleck, S7747, 3 μM), Palbociclib (Selleck, S4482, 1 μM); PD0325901 (MCE, HY-10254); CHIR99021(MCE, HY-10182A), GST tagged GSK-3 beta Protein (MCE, HY-P702734).

### Cell treatment and western blot

mESCs were subjected to UV irradiation (10 J/m^2^) and harvested 6 h later, or treated with doxorubicin (40 nM, 200 nM), or cisplatin (5 µM), with or without MG132 (1 µM) or flavopiridol (1 µM) for 6h. Cells were lysed in RIPA buffer (Beyotime P0013B) supplemented with 0.5 mM DTT, 0.1% PMSF, 5 mM NaF, and 2 mM Na_3_VO_4_. Protein lysates were quantified by BCA assay (Thermo Fisher Scientific, 23,228), resolved by 10% SDS-PAGE, transferred to nitrocellulose membranes (Amersham, 10600001), and immunoblotted with indicated antibodies. Blots were developed using ECL substrate (Bio-Rad, 1705062).

### Kinase assay

*In vitro* kinase assays were performed as previously described (Fang et al., 2022). Briefly, recombinant wild-type or mutant ESRRB proteins were incubated with purified GSK3β (MCE, HY-P702734) protein in a total volume of 20 µL kinase buffer containing 20 mM HEPES (pH 7.6), 50 mM potassium acetate [K(Ac)], 5 mM magnesium acetate [Mg(Ac)_2_], 50 ng/μL BSA, and 30 µM ATP for 10 min at room temperature. The kinase reactions were terminated by adding 5 µL of 5× SDS sample buffer (20% Ficoll, 10% SDS, 50 mM Tris-HCl, pH 6.8, and 50 mM DTT). Phosphorylated ESRRB was analyzed by Western blot using a phospho-threonine/phospho-serine (p-TP/SP) specific antibody (Abcam, ab9344).

### Ubiquitination assay

Mouse ESCs were maintained in 2i/LIF medium and transfected with HA-ubiquitin expression plasmid using Lipofectamine 2000 (Thermo Fisher Scientific) according to the manufacturer’s instructions. Differentiation was initiated by withdrawing LIF and PD0325901 for 3 days, in the presence or absence of the GSK3β inhibitor CHIR99021 (3 µM). Cells were then lysed in IP lysis buffer (20 mM Tris-HCl pH 7.9, 150 mM NaCl, 20 mM MgCl_2_, 75 mM KCl, 0.5% NP-40, 0.5 M EDTA, 10% glycerol) supplemented with protease inhibitor cocktail (Selleck), 1 mM PMSF, 5 mM NaF, and 2 mM Na_3_VO_4_. Lysates were cleared by centrifugation at 12,000 × g for 15 min at 4 °C. Supernatants were incubated with anti-Flag M2 affinity beads (Sigma-Aldrich) for 2 h at 4 °C with rotation. Beads were washed three times with lysis buffer, and bound proteins were eluted by boiling in 2× SDS loading buffer. Ubiquitinated ESRRB was detected by immunoblotting with anti-HA antibody (Santa Cruz), and total ESRRB was detected with anti-Flag antibody (Sigma).

### Flow cytometry

Cells were dissociated into a single-cell suspension using 2 mM EDTA, and then blocked with FACS buffer (2% FBS in PBS) for 30 min at room temperature. After blocking, cells were incubated with primary antibodies for 1 h at room temperature. At least 20,000 events per sample were acquired on a BD FACSCelesta flow cytometer, and the data were analyzed using FlowJo V10.

### AP staining, teratoma and H&E

mESCs were dissociated into single cells and seeded at a density of 600 cells per well. Cells were then cultured in LIF-deficient medium supplemented with retinoic acid (-LIF + RA) to induce differentiation. Following 6 days of culture, alkaline phosphatase (AP) staining was performed using alkaline phosphatase detection kit (Vector, SK-5100). AP staining procedures were performed as previously described (Dai et al., 2014). Briefly, the medium was aspirated, and cells were washed twice with ice-cold PBS and fixed with 4% formaldehyde in PBS for 15 min at room temperature. After washing three times with PBS, cells were incubated with the AP substrate solution in the dark at room temperature for 30 min. The reaction was stopped by rinsing the wells with distilled water.

Teratoma formation assay was performed as previously described (Fang et al., 2022). Briefly, following resuspension in PBS at 5 × 10^6^ cell/mL,100 µL aliquots 5 × 10^5^ of WT, EKO, EKO-WT, and EKO-5A cells were administered *via* subcutaneous injection into the flanks of female NOG-dKO mice (Yaguchi et al., 2018). Progression of tumor growth was tracked periodically until the defined volume limit (1 cm^3^) was attained. Because EKO-5A-derived tumors grow faster than other genotypes, animals in other groups (WT, EKO, and EKO-WT) were euthanized when EKO-5A growth reaches the volume limit.

H&E staining was performed as previously described (Cardiff et al., 2014). Briefly, sections were deparaffinized in eco-friendly xylene substitute (Beyotime, ST975) for 10 min, replaced with fresh solution for another 10 min, and rehydrated through graded ethanol series (100% ethanol for 5 min, 90% ethanol for 2 min, 80% ethanol for 2 min, 70% ethanol for 2 min) to distilled water. Sections were then stained with hematoxylin for 5–10 min (adjusted according to staining results), rinsed in running tap water for ∼10 min, differentiated in 1% acid alcohol for a few seconds, rinsed again in tap water for 10 min, and counterstained with eosin for 30 s–2 min (adjusted according to staining results). After dehydration through graded ethanol, clearing in xylene substitute, and mounting with coverslips. Images were obtained using Pannoramic MIDI digital slide scanner (3DHISTECH).

## Statistics

Representative results from at least three independent experiments were shown. Data are represented as means ± SD. Statistical comparisons were made by using one-way ANOVA tests with GraphPad Prism 10. The significance of difference was set at P values <0.05. In all figures, * denotes P < 0.05, ** denotes P < 0.01, *** denotes P < 0.001, and **** denotes P < 0.0001.

## Data Availability

The original contributions presented in the study are included in the article/Supplementary Material, further inquiries can be directed to the corresponding authors.
